# Black tea differs from green tea: it suppresses long-term memory formation in *Lymnaea*

**DOI:** 10.1080/19420889.2018.1491245

**Published:** 2018-07-03

**Authors:** Jack Zhang, Emily de Freitas, Ken Lukowiak

**Affiliations:** Hotchkiss Brain Institute, Cumming School of Medicine, University of Calgary, Calgary, AB, Canada

**Keywords:** *Lymnaea*, Black tea, obstruction of long-term memory

## Abstract

Foods, such as Green tea (GT), containing the flavonol, (-)-Epicatechin (Epi), enhance the formation of long-term memory (LTM) when snails are operantly conditioned in that substance. That is, a single 0.5 h training session results in LTM; whereas similar training in pond water does not result in LTM. It was of interest to determine if Black tea (BT), which is a more popular beverage than GT and which is derived from the same tea leaves, also enhances LTM formation. We found that BT, unlike GT, depressed homeostatic aerial respiratory behaviour and obstructed LTM formation. We used two different methods to determine if BT altered LTM formation and both procedures showed us that BT obstructed LTM formation. We conclude that BT obstructs LTM formation and depresses homeostatic aerial respiration

Understanding the “how and why” of memory formation is of enormous heuristic interest as it is memory that “makes me, me and you, you”. Memory formation is susceptible to alteration by environmental factors, such as stress and lifestyle choices [1]. One of these key lifestyle choices is diet. We have recently shown [2] that food substances rich in flavonols, especially (-)-Epicatechin (Epi), for example green-tea (GT), enhance memory formation in the fresh water pond snail, *Lymnaea stagnalis*. Seeing how GT enhanced memory formation, we were immediately interested in whether the more popular drink, black tea (BT) has a similar memory enhancing effect.

What is BT? Both GT and BT come from the same plant (*Camellia sinensis*) but to make BT the tea leaves go through an oxidation process called “fermentation” while GT does not [3,4]. This process causes the tea leaves to blacken, possibly to maintain its better flavor far longer than for GT. However, this process substantially reduces (6.16 mg/100 g to 0.49 mg/100 g) the Epi content in BT [5,6]. BT, is the second most widely consumed beverage in the world after water. Thus, BT’s possible impact on cognitive functioning is important to know [4]. BT contains more caffeine than GT, less Epi content; but substantially more thearubigins and theaflavins than GT. These flavan-3-ols may alter cognition [6]. Reports indicate BT consumption increases alertness, possibly due to its caffeine content [4,7,8]. In invertebrates, such as bees, caffeine in some situations enhances memory formation [9]; however, in *Drosophila* caffeine reduces the ability of the flies to form memory on an aversive learning task [10]. Finally, in land snails, a caffeine injection altered the rate of learning [11].

Since *Lymnaea* respond positively to GT as regards to the augmentation of cognitive ability [2], we were interested to know if BT similarly altered cognition (i.e. learning and memory). Reports in the literature show that BT increases alertness, which based on the Yerkes/Dodson/Hebb curve [12] should result in enhanced LTM formation. We hypothesize that BT will enhance LTM formation.

We used the W-strain adult *Lymnaea* (25 mm spine height), which exhibits the “average” memory phenotype [2]. Thus, to cause LTM formation (i.e. a 24 h memory) two 0.5h training sessions separated by a 1h interval are necessary. The W- strain has been used extensively in our laboratory and is the same strain used in the previous experiments testing the effects of Epi and Epi-containing foods on cognition in *Lymnaea* [2,13–16]. In this strain a single 0.5h TS does not result in LTM formation.

For the BT experiments, we used “Twinings English Breakfast Pure Black Tea” purchased from a local national chain grocery store. We prepared the BT for experimentation with two methods: 1) As we previously did with GT [2], by boiling 250 ml pond water (PW) and then steeping the BT bag in PW for 2 minutes. The tea bag was then removed and the tea was left to cool to room temperature (~ 20°C). Once cooled, 100 ml of BT was added to 400 ml of PW. This is denoted as the 1:4 BT; 2) In a similar manner we added 33 ml of cooled BT to 477 ml of pond water. This is denoted as the 1:14 BT.

We measured total breathing time (TBT, the time in seconds that the snail has its pneumostome open) in a 0.5 h observation session. The PW is made hypoxic by bubbling 100% N2 through 500 ml of PW in a 1l beaker for 20 min [17]. The TBT in normal PW is determined before and after a similar session in hypoxic BT. Each session separated by 24h.

To operantly condition aerial respiratory behavior, snails were placed in the hypoxic environment (e.g. BT PW) and every time they attempt to open their pneumostome they received a tactile stimulus to the pneumostome (i.e. an attempted pneumostome opening). The applied tactile stimulus (i.e. poke) caused the pneumostome to close. Snails received either: 1) A Pre-obs TBT session, then 24 h later a single 0.5 h training session (TS) in BT and 24 h later a Post-obs TBT session; 2) Two 0.5 h training sessions separated by 1h. LTM was tested 24 h later. For a more detailed description of the procedures used to operantly condition snails see [12–18].

We found that homeostatic aerial respiratory behaviour triggered by hypoxia was significantly decreased at both concentrations of BT compared to both the pre- and post-observation sessions (Table 1). There was also complete recovery from the depressive effects of the BT on respiration. We conclude that BT at the two concentrations used here cause a depression of homeostatic aerial respiratory behaviour. This result is different from what was found with GT, as GT did not depress aerial respiration [2]. Further experimentation will be necessary to determine why there is respiratory depression in BT.

**Table 1.** 

BT composition	Pre-obs (sec)	BT-obs (sec)	Post-obs (sec)
BT 1:4	281.9	135.2 **	281.8
BT 1:14	393.4	232.1 **	364.7

We used two dilutions on black tea (BT) a 1:4 and a 1:14 BT:PW. The total breathing time (TBT) in the three 0.5h hypoxic observations sessions (Pre-, BT- and Post-obs) sessions are given in seconds for both BT dilutions. For the 1:4 BT dilution a one-way ANOVA 1:4 F_(2, 54)_ = 6.656; p = 0.0026 showed there was a significant effect. The Tukey’s *post-hoc* test indicated that the TBT in BT was significantly less than in either the Pre- or Post-obs sessions. Finally, the Pre- and Post-obs sessions were not different from each other. In a similar manner we analyzed the data for the 1:14 BT dilution. A one-way ANOVA F_(2, 39)_ = 3.171 = 6.761; p = 0.0043; showed there was a significant effect. The Tukey’s *post-hoc* test indicated that the TBT in BT was significantly less than in either the Pre- or Post-obs sessions. Finally, the Pre- and Post-obs sessions were not different from each other.

Because we found that BT caused respiratory depression we first used a procedure previously employed [17] where we first measure TBT in a Pre-obs session and then measure TBT 24h after a single 0.5 h training session (i.e. the Post-obs session). We used this procedure with both green tea (GT, at the concentration (1:4) used previously [2]) to validate this method and with BT at the 1:4 concentration. These data are presented in Figure 1. When snails were trained in GT the total breathing time (TBT) in the Post-obs session was statistically significantly lower than in the Pre-obs session (Figure 1(a)). Thus, training in GT resulted in LTM formation. On the other hand, when we trained in BT at the 1:4 concentration (Figure 1(b)) we saw no significant difference in the TBT between the Pre- and Post-obs sessions, indicating that LTM had not formed.

**Figure 1.**
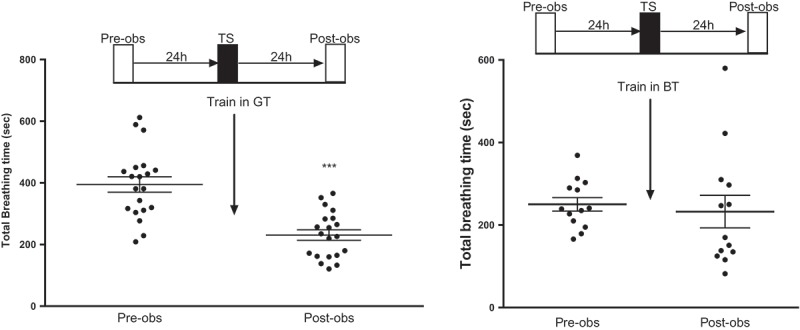
Green tea (GT) enhances LTM formation but BT does not. A) In a naïve cohort (n = 20) of snails total breathing time (TBT) for a 0.5 h hypoxic session in pond water was first measured (Pre-obs session). These snails 24 h later then received a 0.5h training session (TS) in GT. Twenty-four hours after the TS the TBT in these snails was again measured (Post-obs). A paired t-test was performed on these data (t = 5.597, df = 19; p < 0.001) and showed that the TBT in the Post-obs session was significantly less than in the Pre-obs session showing that LTM had formed following the single 0.5 h TS. B) A naïve cohort of snails (n = 13) received a similar procedure described in A, except that the training was performed in BT rather than GT. A paired t-test (t = 0.4570, df = 12; p + 0.6558) indicated that the TBT in the Post-obs sessions was not significantly different that the Pre-obs session indicating that LTM had not formed.

We then asked whether if we trained in BT using the typical two 0.5 h training sessions separated by a 1h interval and then testing for LTM 24h later [12–18] would LTM form? Using this procedure GT was shown to enhance or to reverse a blockade of LTM formation [13–15]. Here we found (Figure 2) that training in the 1:14 BT blocked LTM formation. That is, the number of attempted pneumostome openings in the memory test session (MT) was not significantly less than the number in TS1. In the W-strain snails used here, this training procedure results in LTM formation when the training and testing sessions take place in pond water [1,18].

Previously we showed that a natural food substance, Epi, that is present in a number of foods was able to enhance LTM formation if the snail was trained in Epi-PW or was placed in Epi-PW during the consolidation period following training in PW [13–15,19]. More recently we showed that LTM enhancement also occurs when snails are trained in food substances (e.g. GT, cocoa, and apple peel pond water) that contain high levels of Epi [2]. It has not yet been tested whether placing snails in these foods immediately after training (i.e. the consolidation period) enhances LTM formation. As both BT and GT come from the same plant it was of interest to determine if BT also enhanced LTM formation; especially since BT is more often imbibed than GT.

Two unanticipated findings emerged: 1) BT causes a depression of homeostatic aerial respiratory behaviour (Table 1); and 2) Training in BT not only does not enhance LTM formation but prevents LTM formation (Figures 1 and 2). We are uncertain what substance(s) in BT causes these effects on respiratory behaviour. Typically, depending on brewing time and grade of the tea leaves used, BT has up to 3-times the amount of caffeine than does GT [20,21]). Here we kept brewing times similar between the GT and BT “brews” so we expect that the caffeine is higher in BT than it is in the GT used. However, preliminary data show that caffeine by itself does not bring about similar effects. We also suspect that because we used a brand and type (teabags) sold in a large supermarket chain that the highest quality leaves may not have been in this tea [3]. However, the respiratory depression seen here is in contrast to what we expected; as caffeine typically increases respiration and is in fact the drug of choice for treating human infant apnea [22,23]. Further study will be necessary to determine what substance in BT causes this depression [24].

**Figure 2.**
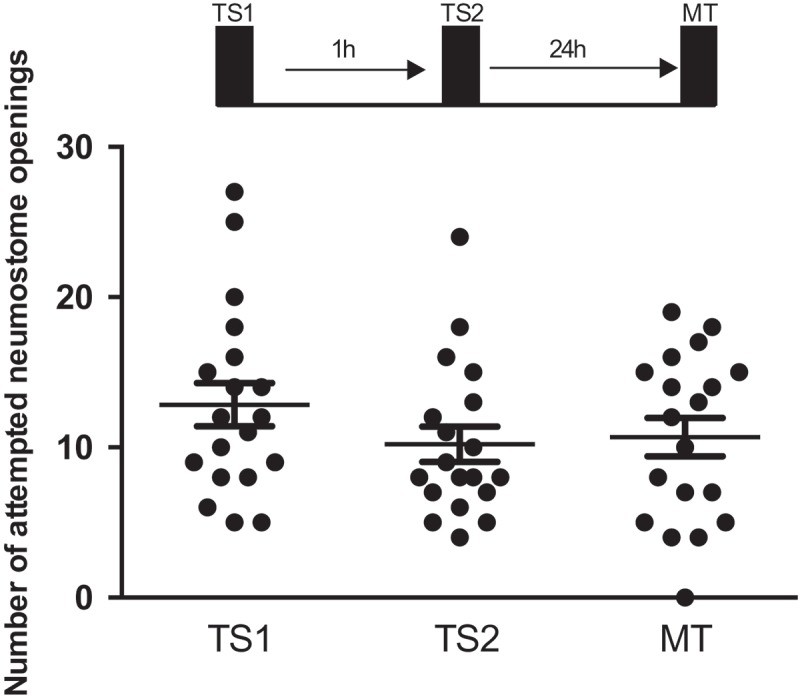
BT blocks LTM formation. A naïve cohort of snails (n = 19) received two 0.5h training sessions separated by a 1h interval (TS1 and TS2) and then memory was tested 24h later (MT). The training and memory test sessions were conducted in the 1:14 BT pond water. A one-way ANOVA was performed on these data (F_(2,36)_ = 2.219; p = 0.1234) which indicated that there was no significant effect. This was confirmed by A Tukey’s *post-hoc* test which showed that there was not a significant difference in the number of attempted openings between any of the training (TS1 and TS2) or memory test (MT) sessions. Thus, the criteria for LTM formation were not met.

We were surprised that BT not only did not enhance LTM formation but actually prevented its formation (Figure 2). It has been shown that BT has positive effects on cognition [7–9] primarily by enhancing attention. The effects of BT on cognition may also be due to what we call the Yerkes-Dodson/Hebb (Y-D/H) curve effect [12]. The Y-D/H is an inverted-U function and simply stated the Y-D/H curve posits that there is an optimal level of arousal that best suits memory formation. If BT causes the subject to move to this optimal portion of the curve memory formation will be enhanced; but if it moves the subject to the less optimal portions of the function memory will be more difficult to make. It is possible here, that while we used the same “dilution” factor in making BT-PW as we did with GR [2] the substances in BT that are not in GT at this concentration move the snails too much from the optimal part of the function. It is worth noting, however, that the effects of drinks containing caffeine (e.g. BT and coffee) on memory formation and recall are inconsistent across many studies [7–11]. We will have to perform more experiments on this using different dilutions of BT in the PW.

In conclusion that changes in aerial respiratory behaviours brought about by snails experiencing BT are significantly different than those caused by GT or other food substances previously tested. In our hands BT does not enhance LTM formation.
